# Supplementary material for: Heterogeneity in establishment of polyethylene glycol-mediated plasmid transformations for five forest pathogenic Phytophthora species
Source: PLoS One. 2024 Sep 10;19(9):e0306158. doi: 10.1371/journal.pone.0306158 (PMC11386421; doi:10.1371/journal.pone.0306158)
Supplement: S1 Appendix — Details of the recipes for all culture media and transformation solutions used in this study. (DOCX) [file pone.0306158.s001.docx]

# S1 Appendix: Supplementary Methods

## Geneticin MICs

The table below summarizes the geneticin (G418) concentrations tested to determine the minimum inhibitory concentration (MIC) for each *Phytophthora* species used in this study.

| **Species** | **Isolate(s) Tested** | **G418 Concentration Gradient (μg/mL)** |
| --- | --- | --- |
| *Phytophthora cactorum* | Larch FF-42 2Pa | 0, 2, 5, 10, 20, 30, 50 |
| *Phytophthora cinnamomi* | CBS 270.55 | 0, 2, 5, 10, 20, 30, 40, 50 |
| *Phytophthora cryptogea* | RS-2006-soil-d8 | 0, 2, 5, 10, 20, 30, 50 |
| *Phytophthora ramorum* | NA2 16_0386_0016, NA2 17_0134_0030 | 0, 2, 5, 10, 20, 30, 40, 50 |
| *Phytophthora sojae* | P6497 (R2) | 0, 2, 5, 10, 20, 30, 40, 50 |
| *Phytophthora syringae* | Alder 12 | 0, 2, 5, 10, 20, 30, 50 |

## Growth Media

All recipes for growth media and transformation solutions are adapted from:

Fang Y, Cui L, Gu B, Arredondo F, Tyler BM (2017) Efficient Genome Editing in the Oomycete *Phytophthora sojae* Using CRISPR/Cas9. Current Protocols in Microbiology 44:21A.1.1-21A.1.26

Abbreviations:

| dH_2_O | distilled water |
| --- | --- |
| × *g* (rcf) | g-force (relative centrifugal force) |
| g | grams |
| mL | millilitres |
| M | molar (moles/litre) |
| rpm | revolutions per minute |

### 5X V8 Medium

| **Component** | **Amount** |
| --- | --- |
| V8 juice | 340 mL |
| Calcium carbonate (CaCO_3_) | 5 g |
| Centrifuged at 3220 × *g* (~4000 rpm) for 15 minutes to clarify; supernatant retained. Not autoclaved. Stored in the fridge (~4°C) for up to three months. | |

### 1X (20%) V8 Broth/Agar (V8B/V8A)

| **Component** | **Amount** |
| --- | --- |
| 5X V8 medium | 200 mL |
| dH_2_O | 800 mL |
| Agar (for V8A) | 15 g (1.5%) |
| Autoclaved (40-minute liquid cycle) and stored in the fridge (~4°C) until used. | |

### Basic Pea Broth (PB)

| **Component** | **Amount** |
| --- | --- |
| Frozen sweet peas | 120 g |
| dH_2_O | 800 mL |
| Autoclaved (40-minute liquid cycle) and filtered through four layers of cheesecloth. Used as base for pea-mannitol and nutrient pea media (see recipes below). | |

### Pea-0.5 M Mannitol (PM) & V8-0.5 M Mannitol (VM) Medium

| **Component** | **Amount** |
| --- | --- |
| D-mannitol | 91.1 g |
| Calcium carbonate (CaCO_3_) | 2 g |
| Calcium chloride (CaCl_2_) | 1 g |
| Basic pea broth for PM -*or*-  1X V8 medium for VM | 800 mL |
| Centrifuged at 3220 × *g* (~4000 rpm) for 10 minutes to clarify; supernatant retained. Volume brought to 1000 mL with dH_2_O, and agar added (1% or 1.5%) for solid medium. Autoclaved (40-minute liquid cycle) and stored in the fridge (~4°C) until used. | |

### Nutrient Pea Broth/Agar (NPB/NPA) & Nutrient V8 Broth/Agar (NVB/NVA)

| **Component** | **Amount** |
| --- | --- |
| D-glucose | 5.0 g |
| D-mannitol | 5.0 g |
| D-sorbitol | 5.0 g |
| Potassium nitrate (KNO_3_) | 3.0 g |
| Yeast extract | 2.0 g |
| Calcium carbonate (CaCO_3_) | 2.0 g |
| Potassium dihydrogen phosphate (KH_2_PO_4_) | 1.0 g |
| Dipotassium hydrogen phosphate (K_2_HPO_4_) | 1.0 g |
| Magnesium sulphate (MgSO_4_) | 0.5 g |
| Calcium chloride (CaCl_2_) | 0.1 g |
| Basic pea broth for NPB -*or*-  1X V8 medium for NVB | 800 mL |
| Trace elements^*^ | 2.0 mL |
| Vitamin stock^*^ | 2.0 mL |
| Centrifuged at 3220 × *g* (~4000 rpm) for 10 minutes to clarify; supernatant retained. Volume brought to 1000 mL with dH_2_O, and agar added (1.5%) for solid medium. Autoclaved (40-minute liquid cycle), trace elements and vitamin stock added, and final medium stored in the fridge (~4°C) until used. | |

^*^Added after autoclaving – see recipes below

#### Trace Elements:

| **Component** | **Amount** |
| --- | --- |
| Iron (III) citrate trihydrate (FeC_6_H_5_O_7_•3H_2_O) -*or*-  *Iron (III) citrate (FeC_6_H_5_O_7_)* | 0.215 g  *0.176 g* |
| Zinc sulphate heptahydrate (ZnSO_4_•7H_2_O) | 0.150 g |
| Copper sulphate pentahydrate (CuSO_4_•5H_2_O) | 0.030 g |
| Manganese sulphate monohydrate (MnSO_4_•H_2_O) | 0.015 g |
| Boric acid (H_3_BO_3_) | 0.010 g |
| Molybdenum trioxide (MoO_3_) | 0.007 g |
| Milli-Q^Ⓡ^ water | 400 mL |
| Stirred until dissolved. The final solution is filter sterilized, wrapped in aluminum foil (light sensitive) and stored at 4°C for up to 1 year. | |

#### Vitamin Stock:

| **Component** | **Amount** |
| --- | --- |
| Thiamine-HCl | 0.3800 g |
| Pyridoxine-HCl | 0.1800 g |
| Nicotinic acid | 0.0600 g |
| Riboflavin | 0.0150 g |
| I-inositol (myo-inositol) | 0.0120 g |
| Biotin | 0.0002 g |
| Folic acid | 0.0002 g |
| Coconut milk (*optional*) | 50 mL |
| Milli-Q^Ⓡ^ water | 300 mL |
| Stirred until dissolved. The final solution is filter sterilized, wrapped in aluminum foil (light sensitive) and stored at 4°C for up to 1 year. | |

## Transformation Solutions

### 0.8 M Mannitol

| **Component** | **Amount** |
| --- | --- |
| D-mannitol | 145.76 g |
| Milli-Q^Ⓡ^ water | 700 mL |
| Stirred until dissolved. Volume brought to 1000 mL with Milli-Q^Ⓡ^ water, stirred, and split into 250 mL aliquots. Autoclaved (40-minute liquid cycle) and stored at room temperature until used. | |

### 0.5 M CaCl_2_

| **Component** | **Amount** |
| --- | --- |
| Calcium chloride (CaCl_2_) | 7.35 g |
| Milli-Q^Ⓡ^ water | 70 mL |
| Stirred until dissolved. Volume brought to 100 mL with Milli-Q^Ⓡ^ water and stirred. Filter sterilized and stored at room temperature until used. | |

### 1 M KOH

| **Component** | **Amount** |
| --- | --- |
| Potassium hydroxide (KOH) | 14.03 g |
| Milli-Q^Ⓡ^ water | 200 mL |
| Stirred until dissolved. Volume brought to 250 mL with Milli-Q^Ⓡ^ water and stirred. Stored at room temperature (used to adjust pH of MES solution). | |

### 0.5 M MES KOH

| **Component** | **Amount** |
| --- | --- |
| 4-Morpholineethanesulfonic acid (MES) | 4.88 g |
| Milli-Q^Ⓡ^ water | 40 mL |
| Stirred until dissolved, pH adjusted to 5.7 using 1 M KOH. Volume brought to 50 mL with Milli-Q^Ⓡ^ water and stirred. Filter sterilized and stored at room temperature until used. | |

### 0.5 M KCl

| **Component** | **Amount** |
| --- | --- |
| Potassium chloride (KCl) | 1.86 g |
| Milli-Q^Ⓡ^ water | 40 mL |
| Stirred until dissolved. Volume brought to 50 mL with Milli-Q^Ⓡ^ water and stirred. Filter sterilized and stored at room temperature until used. | |

### PEG (40% v/v) Solution

| **Component** | **Amount** |
| --- | --- |
| Polyethylene glycol (PEG) 4000 | 24 g |
| 0.8 M mannitol | 15 mL |
| 0.5 M CaCl_2_ | 12 mL |
| Milli-Q^Ⓡ^ water | 12 mL |
| Stirred to dissolve and filter sterilized. Used immediately (can be stored at 4°C for a maximum of two weeks). | |

### Enzyme Solution

| **Component** | **Amount** |
| --- | --- |
| Lysing enzymes from *Trichoderma harzianum*^*^ | 0.2 g |
| Cellulase^✢^ | 0.2 g |
| 0.8 M mannitol | 20 mL |
| 0.5 M KCl | 1.6 mL |
| 0.5 M MES KOH (pH 5.7) | 1.6 mL |
| 0.5 M CaCl_2_ | 0.8 mL |
| Volume brought to 40 mL Milli-Q^Ⓡ^ water. Final solution filter sterilized and stored at 4°C until use (maximum one day). | |

^*^Lysing Enzymes from *Trichoderma harzianum* (Sigma catalog #: L1412)

^✢^CELLULYSIN® Cellulase, *Trichoderma viride* (Calbiochem catalog #: 219466)

### W5 Solution

| **Component** | **Amount** |
| --- | --- |
| Glucose | 7.8 g |
| Calcium chloride (CaCl_2_) | 4.6 g |
| Sodium chloride (NaCl) | 2.25 g |
| Potassium chloride (KCl) | 0.093 g |
| Milli-Q^Ⓡ^ water | 200 mL |
| Stirred until dissolved. Volume brought to 250 mL with Milli-Q^Ⓡ^ water. Final solution split into 50 mL aliquots, autoclaved (40-minute liquid cycle), and stored at 4°C for a maximum of six months. | |

### MMg Solution

| **Component** | **Amount** |
| --- | --- |
| D-mannitol | 18.22 g |
| Magnesium chloride hexahydrate (MgCl_2_•6H_2_O) | 0.76 g |
| 0.5 M MES KOH (pH 5.7) | 2 mL |
| Milli-Q^Ⓡ^ water | 200 mL |
| Stirred until dissolved. Volume brought to 250 mL with Milli-Q^Ⓡ^ water. Final solution split into 50 mL aliquots, autoclaved (40-minute liquid cycle), and stored at 4°C for a maximum of six months. | |
