# Supplementary figures and images for: Heterogeneity in establishment of polyethylene glycol-mediated plasmid transformations for five forest pathogenic Phytophthora species
Source: PLoS One. 2024 Sep 10;19(9):e0306158. doi: 10.1371/journal.pone.0306158 (PMC11386421; doi:10.1371/journal.pone.0306158)

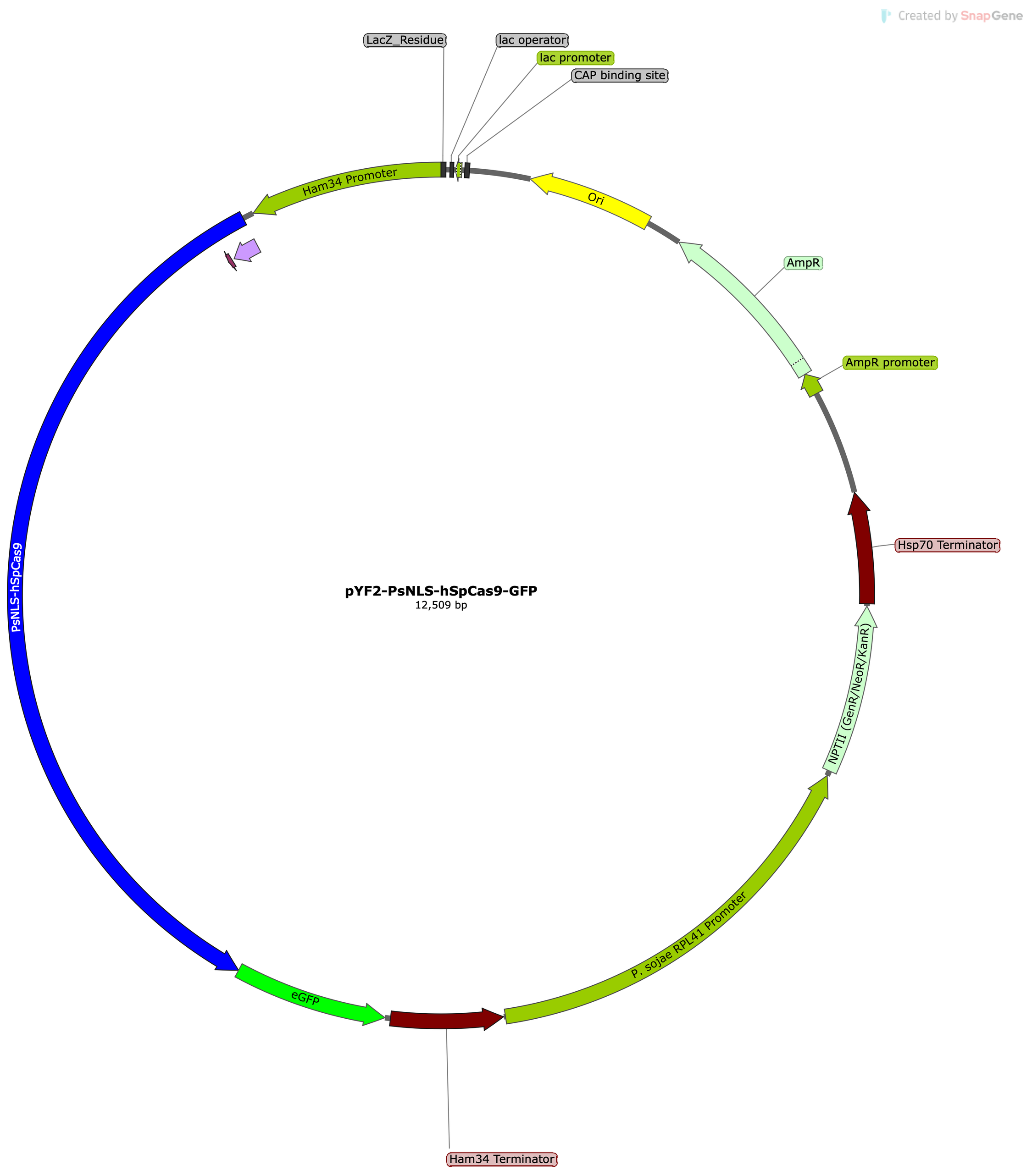

Supplement: S1 Fig — Map of the pYF2-PsNLS-hSpCas9-GFP (abbreviated pYF2-PsCG) transformation plasmid developed by Fang and Tyler (2016) for expression of hSpCas9 in Phytophthora sojae. The plasmid sequence was determined from Fang and Tyler (2016) and Fang et al. (2017) and is provided in FASTA format in S1 File. Plasmid map created using SnapGeneⓇ Viewer software v. 7.0.1 (from Dotmatics; available at snapgene.com). (TIF) [file pone.0306158.s006.tif]

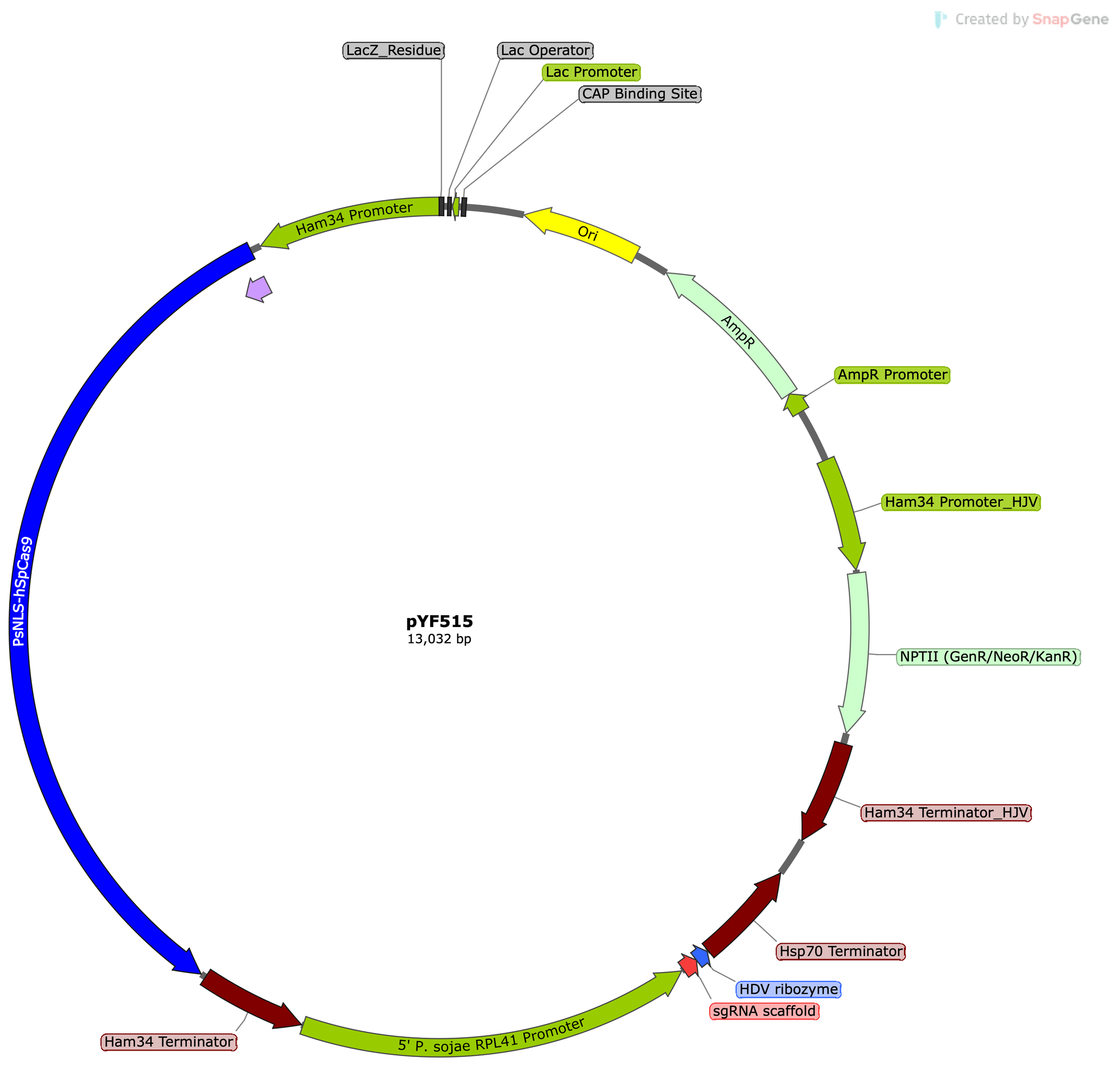

Supplement: S2 Fig — Map of pYF515, the ’all-in-one’ plasmid developed by Fang et al. (2017) for expression of hSpCas9 and a single-guide RNA (sgRNA) for CRISPR/Cas9 gene editing. The plasmid sequence was supplied by Dr. Felipe Arredondo (Oregon State University) and is provided in FASTA format in S2 File. Plasmid map created using SnapGeneⓇ Viewer software v. 7.0.1 (from Dotmatics; available at snapgene.com). (TIF) [file pone.0306158.s007.tif]

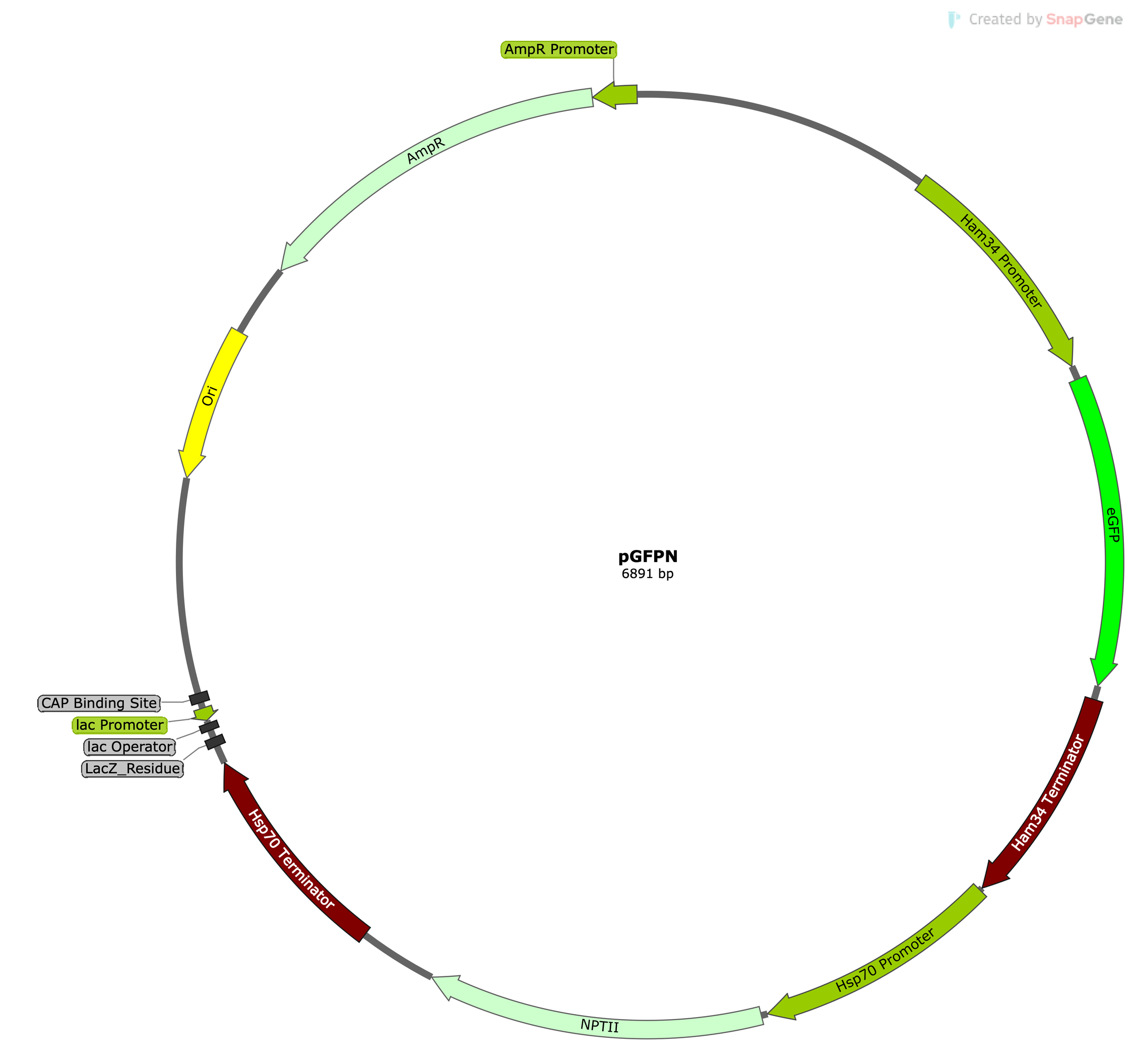

Supplement: S3 Fig — Map of the transformation plasmid pGFPN developed by Ah-Fong and Judelson (2011). The plasmid sequence was supplied by Dr. Felipe Arredondo (Oregon State University) and is provided in FASTA format in S3 File. Plasmid map created using SnapGeneⓇ Viewer software v. 7.0.1 (from Dotmatics; available at snapgene.com). (TIF) [file pone.0306158.s008.tif]

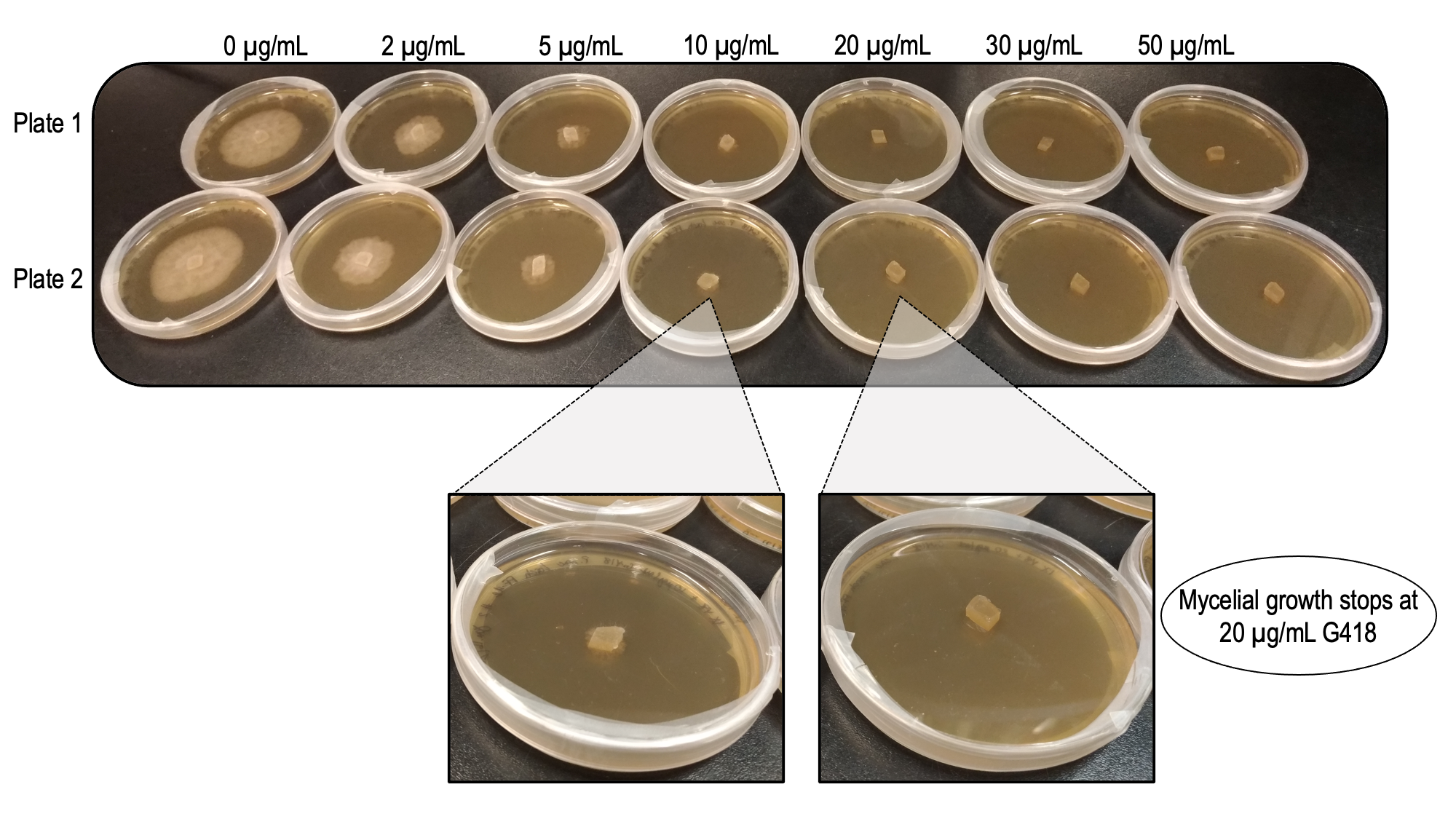

Supplement: S4 Fig — An example of the P. cactorum isolate Larch FF-42 2Pa growth assay demonstrating how the geneticin (G418) minimum inhibitory concentration (MIC) was determined for each forest Phytophthora species in this study. Each isolate was grown on V8 agar supplemented with a gradient of G418 concentrations (labels above the image), and two replicate plates were tested for each concentration (labels to the left of image). The first G418 concentration at which mycelial growth stopped was recorded as MIC for that species/isolate. (TIF) [file pone.0306158.s009.tif]

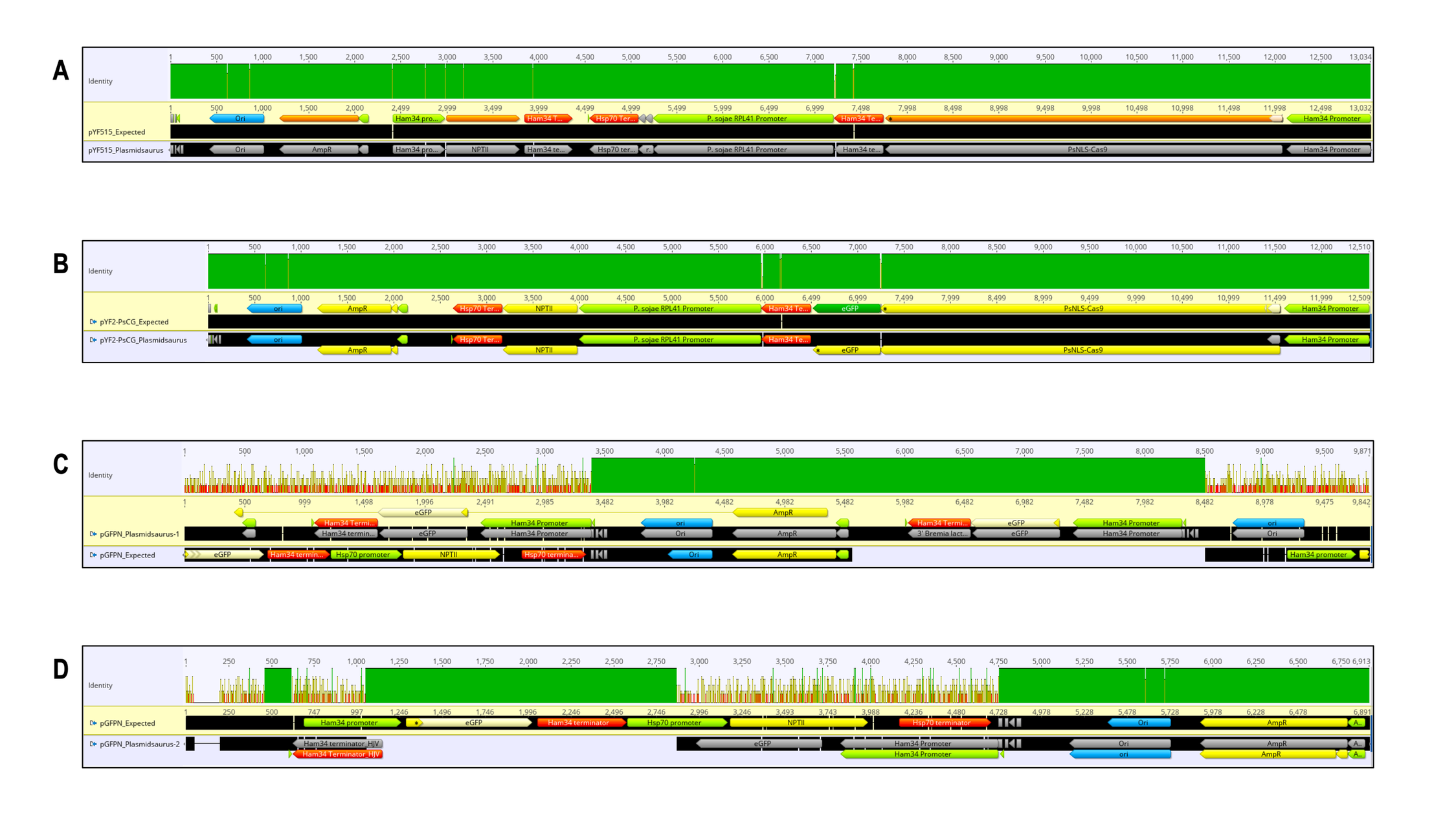

Supplement: S5 Fig — A) Paired alignment of the expected pYF515 sequence (top) and the Plasmidsaurus pYF515 sequence (bottom). The pairwise identity of the two sequences is 99.8% with minor differences in sequence in the nptII gene and its ham34 promoter, both the ham34 terminators, and in the origin of replication (ori). B) Paired alignment of the expected pYF2-PsCG sequence (top) and the Plasmidsaurus pYF2-PsCG sequence (bottom). The pairwise identity of the two sequences is 99.8% with minor differences in sequence at the end of the Cas9 gene, in the ham34 terminator, and in the origin of replication (ori). C) Paired alignment of the Plasmidsaurus pGFPN (first plasmid prep) sequence (top) and the expected pGFPN sequence (bottom). The pairwise identity of the two sequences is 52.9%, with sequence inversions, repeats, and deletion of the nptII gene in the Plasmidsaurus sequence (9,842 bp). D) Paired alignment of the expected pGFPN sequence (top) and the Plasmidsaurus pGFPN (second plasmid prep) sequence (bottom). The pairwise identity of the two sequences is 62.6% with sequence inversions, and a deletion of the nptII gene in the Plasmidsaurus sequence (4,927 bp). All four alignments were generated in Geneious Prime 2023.2.1 (https://www.geneious.com) using the Geneious assembler (Plasmidsaurus sequence mapped to expected sequence). (TIF) [file pone.0306158.s010.tif]
