# Supplementary material for: Heterogeneity in establishment of polyethylene glycol-mediated plasmid transformations for five forest pathogenic Phytophthora species
Source: PLoS One. 2024 Sep 10;19(9):e0306158. doi: 10.1371/journal.pone.0306158 (PMC11386421; doi:10.1371/journal.pone.0306158)
Supplement: S1 Raw image — (PDF) [file pone.0306158.s011.pdf]

Raw image used for manuscript Fig 2

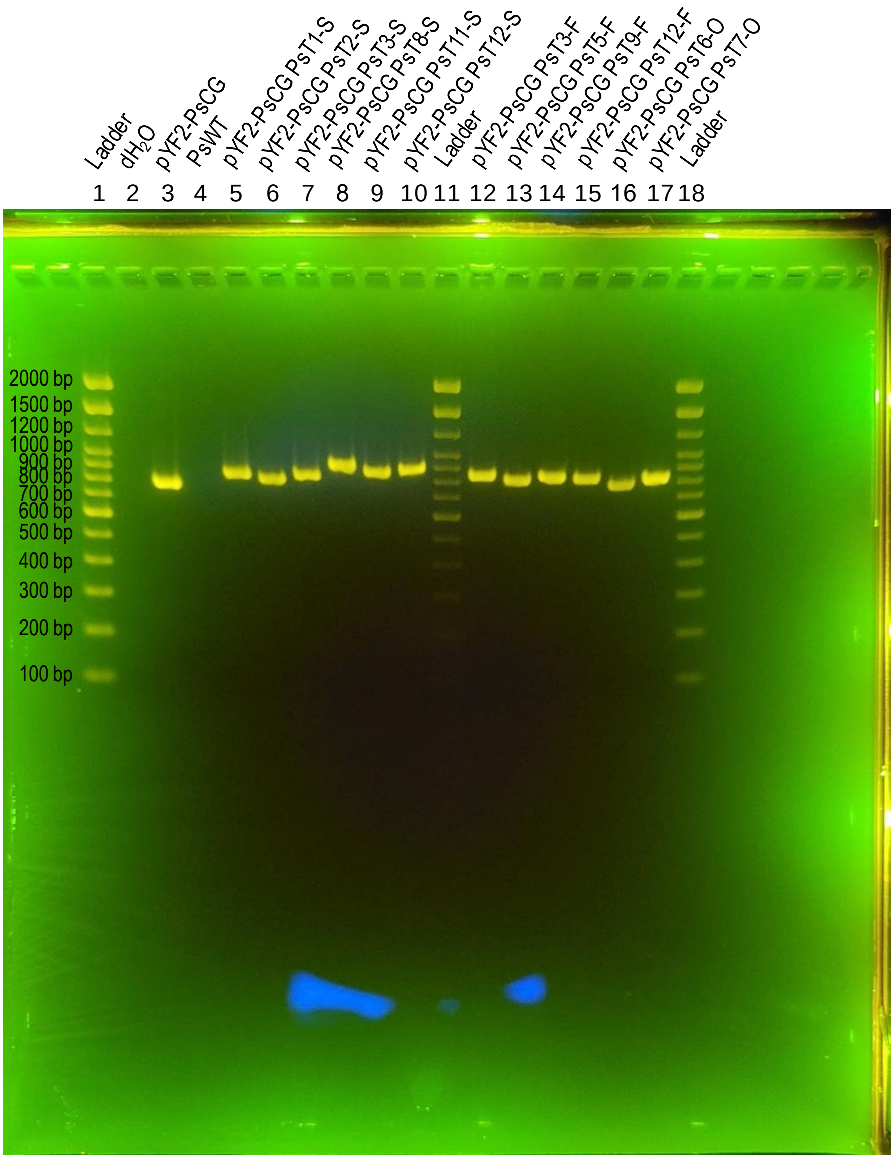

Method: Gel was run at 100 volts for 75 minutes on 1.75% agarose in 0.5X TBE buffer with a 100 bp DNA Ladder (Thermo Fisher Scientific, Waltham, MA, USA). All samples were loaded with Safe-Green™ stain (Applied Biological Materials Inc., Richmond, BC, Canada) at a ratio of 1 µL Safe-Green: 2 µL water: 3 µL PCR product. The gel was visualized using an PI-1002 PrepOne™ Sapphire Blue LED Illuminator (Embi Tec, San Diego, CA, USA), and the image was taken on a Samsung Galaxy S20 FE phone using a light box.

Raw image used for manuscript Fig 4

***Note:** the three "X" samples on the right of the gel were not included because they were from a completely unrelated experiment. A master's student in the lab was troubleshooting a fungal ITS PCR and ran the samples on this gel to see what their products looked like.

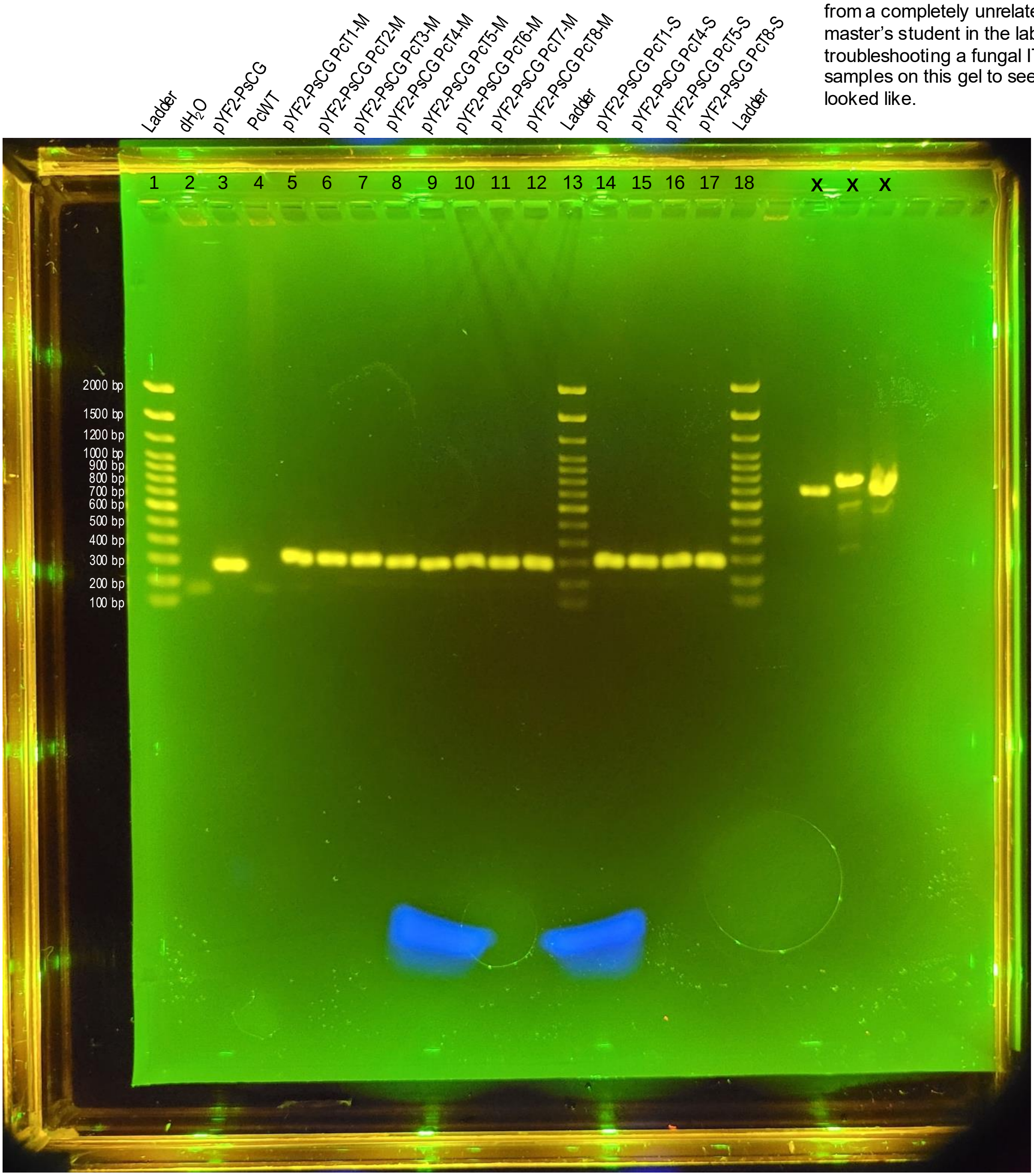

Method: Gel was run at 100 volts for 60 minutes on 1% agarose in 0.5X TBE buffer with a 100 bp DNA Ladder (Thermo Fisher Scientific, Waltham, MA, USA). All samples were loaded with Safe-Green™ stain (Applied Biological Materials Inc., Richmond, BC, Canada) at a ratio of 1 µL Safe-Green: 5 µL PCR product. The gel was visualized using an PI-1002 PrepOne™ Sapphire Blue LED Illuminator (Embi Tec, San Diego, CA, USA), and the image was taken on a Samsung Galaxy S20 FE phone using a light box.

Raw image used for manuscript Fig 6

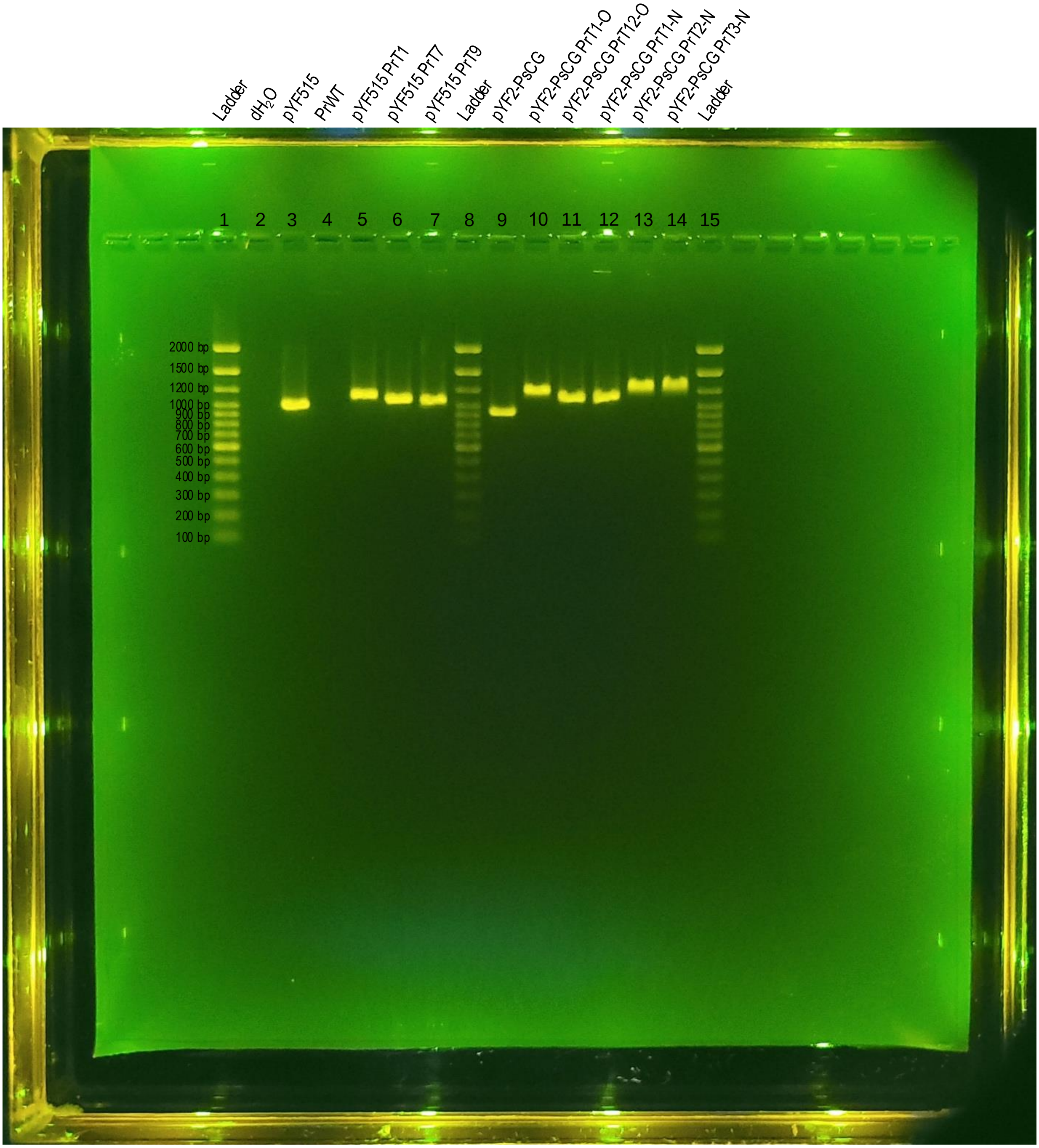

Method: Gel was run at 50 volts for 90 minutes on 1.25% agarose in 0.5X TBE buffer with a 100 bp DNA Ladder (Thermo Fisher Scientific, Waltham, MA, USA). All samples were loaded with Safe-Green™ stain (Applied Biological Materials Inc., Richmond, BC, Canada) at a ratio of 1 µL Safe-Green: 4 µL water: 1 µL PCR product. The gel was visualized using an PI-1002 PrepOne™ Sapphire Blue LED Illuminator (Embi Tec, San Diego, CA, USA), and the image was taken on a Samsung Galaxy S20 FE phone using a light box.
