# Supplementary material for: Heterogeneity in establishment of polyethylene glycol-mediated plasmid transformations for five forest pathogenic Phytophthora species
Source: PLoS One. 2024 Sep 10;19(9):e0306158. doi: 10.1371/journal.pone.0306158 (PMC11386421; doi:10.1371/journal.pone.0306158)
Supplement: Attachment — Submitted filename: Response to Reviewers.pdf [file pone.0306158.s013.pdf]

Dear PLOS ONE Editor and Reviewers,

We, the authors of “Heterogeneity in establishment of polyethylene glycol-mediated plasmid transformations for five forest pathogenic *Phytophthora* species”, would like to thank all of you for taking the time to provide constructive feedback on our manuscript. Below, we have addressed each of your comments point-by-point. Editor and reviewer comments are numbered with our responses following in point form. Any page and line numbers we reference correspond to the revised manuscript we are submitting (“Revised Article with Changes Highlighted.docx”).

Editor Comments

1. “*Please ensure that your manuscript meets PLOS ONE's style requirements, including those for file naming*.”
    - We have reviewed the PLOS ONE style documents and have now corrected style errors in the manuscript and file names.

2. “*We noted in your submission details that a portion of your manuscript may have been presented or published elsewhere…Please clarify whether this publication was peer-reviewed and formally published. If this work was previously peer-reviewed and published, in the cover letter please provide the reason that this work does not constitute dual publication and should be included in the current manuscript.*”
    - No, this publication was ***not*** peer-reviewed and formally published. It was simply posted to the public thesis repository of the University of British Columbia, called cIRcle. The thesis can be viewed at this link: https://open.library.ubc.ca/soa/cIRcle/collections/ubctheses/24/items/1.0441005?o=2

3. “*We note that you have included the phrase “data not shown” in your manuscript. Unfortunately, this does not meet our data sharing requirements. PLOS does not permit references to inaccessible data. We require that authors provide all relevant data within the paper, Supporting Information files, or in an acceptable, public repository. Please add a citation to support this phrase or upload the data that corresponds with these findings to a stable repository (such as Figshare or Dryad) and provide and URLs, DOIs, or accession numbers that may be used to access these data. Or, if the data are not a core part of the research being presented in your study, we ask that you remove the phrase that refers to these data.*”
    - Upon reflection of the use of this phrase, we realized that it is not necessary. The data we were referring to are in the S1 Table of the supporting information. We have now edited the manuscript accordingly.

4. “*Please note that Table 2 is missing, but Tables 1, 3, and 4 is included in the manuscript*.”
    - Thank you for catching this, and apologies for the error. We have now corrected the table numbers in the manuscript.

5. “*We are unable to open your Supporting Information file S1_file.fa, S2_file.fa, and S3_file.fa. Please kindly revise as necessary and re-upload.*”
    - We have now converted these fasta files to .txt files in the hope that the text files are more compatible across different operating systems. We will

re-upload the new files with the revised manuscript.

6. "*Please review your reference list to ensure that it is complete and correct. If you have cited papers that have been retracted, please include the rationale for doing so in the manuscript text, or remove these references and replace them with relevant current references. Any changes to the reference list should be mentioned in the rebuttal letter that accompanies your revised manuscript. If you need to cite a retracted article, indicate the article's retracted status in the References list and also include a citation and full reference for the retraction notice.*"
    - We have gone through the reference list to ensure that everything listed is referenced in our manuscript and we found only one minor spelling error in a species name in one of the references we cited - we have now corrected this error in the revised manuscript. We have also looked up all 62 references we cite on Google Scholar and have not found any that have been retracted. We only found one article that has been corrected since its initial publication: Yang *et al.* 2018 Scientific Reports **8**: 6534. However, the correction was minor and did not change the content or scientific findings of the article.

Reviewer #1 Comments:

1. "*L154 Protoplast isolation and transformation – Please provide information on the source of enzymes used in this study.*"
    - We have now provided the sources of the enzymes used in our study in the S1 Appendix document (where we provide the detailed recipe for the protoplast enzyme solution). This new version of the appendix is now a file named "S1_appendix_revised" and has been uploaded with the revised manuscript.

2. "*L170 After three days of growth – The growth rates of Phytophthora strains can vary, and the growth phase and physiological state of the mycelia can affect their transformation competence. Did the authors make any effort to optimize the growth duration for each species?*"
    - This is a very good point. While we did find that there were minor differences in growth rate between isolates, we found with all our isolates that three days produced the optimal amount of mycelial growth that was comparable to the *P. sojae* control which we were aiming to replicate. Optimizing the time grown in liquid medium for each individual isolate is certainly something we would want to explore in future work to determine if younger (or older) mycelium would have any effect on protoplast formation or transformation efficiency.

3. "*L241 Table 2 is missing*"
    - Thank you for pointing this out–we moved Table 2 to the supplementary materials and forgot to rename the subsequent tables. We have now corrected this error.

4. "*L254 pGEPN samples were 9,842 bp and 4,927 bp – Have the authors linearized those plasmids with an appropriate enzyme and estimated the sizes on the agarose gel? If so, were the estimated values consistent with the sequencing results?*"

- Yes, it was actually the strange results we saw in our restriction enzyme digests that led us to pursue sequencing two separate plasmid preparations to try to understand the anomalous results. We performed several restriction enzyme digests in the lab, both with one enzyme for linearized plasmid and with two enzymes to assess the resulting band sizes. The results were anomalous with none of the fragments migrating to the correct size. Both the single and double digests of the 9,842 bp preparation did indeed indicate a larger than expected plasmid. For the 4,927 bp preparation, the restriction enzyme results indicated that the enzymes did not cut, despite trying two different enzymes (BsaI and NheI) that should have cut the pGFPN plasmid. The sequencing results indicate that the recombination of this plasmid resulted in the loss of these restriction enzyme sites.

5. "*L293 PCR performed on DNA from from (delete one)*"
    - Thank you for noting this– we have now corrected this error.

6. "*L295 (expected product size is 232 bp). Why do the sizes of PCR products on lanes 3 and 4 look different? Also, why do all products look equal or larger than 300 bp?*"
    - This is a very good point – the PCR products from the transformation plasmids used in this study consistently yielded somewhat unexpected results. We designed a number of different primers to try to alleviate the problem, but it persisted. In response to this concern from Reviewer #1, we returned to the lab to try different gel electrophoresis conditions to see if that would help resolve the differences in migration pattern that suggests different band sizes. By adjusting the gel conditions, we were able to improve the consistency somewhat, but there were still clearly differences in band size (see new Figure 2). Subsequently, we sent all the products for Sanger sequencing in both the forward and reverse directions. The results indicate that the products are all identical in sequence (this aligns with what we have found in the past with PCRs done on the pYF2-PsCG plasmid). Given these sequencing results, we expect that the differences in band size are potential secondary structures formed or artifacts of the PCR reaction itself. However, given that in this study, we were using the diagnostic PCRs simply for the presence/absence of a band associated with plasmid DNA, and we found that the negative control had a clear absence of the band, while the positive plasmid controls and the positive transformants all had a clear presence of the band, we think that these gel electrophoresis results still clearly demonstrate the presence of plasmid DNA in transformants. We have now updated Fig 2 accordingly, and also added a note in the Results to address the differences in band size (L280-L286). We also added a similar note to address the different band sizes in Fig 6 (L347-L350).

7. "*L362 Of the five forest Phytophthora species we tested…One or two isolates were tested for each species. Is there any research indicating that variations in transformation efficiency between species are greater than those within species? It's possible that testing eight isolates of P. cactorum could show a similar level of variation in transformation efficiency observed in this study.*"
    - This is an excellent point, and something that we try to address in the Discussion (L421-422, L434-438, L451-452). It is absolutely possible that

testing more isolates of *P. cactorum* would show similar levels of variation in transformation efficiency. While this is something we would be keen to explore in future studies, it was outside of the scope of the current study. We have now also acknowledged this limitation to our study in the Discussion (L516-518).

8. "*L431 ... makes this explanation somewhat less likely. Can you provide a p-value?*"
   - This statement in the Discussion was not made using statistical analyses, therefore, we cannot provide a p-value. We were simply trying to compare the likelihood of possible explanations for transformation failure in *P. cinnamomi* based on results from previous studies. We have now modified the sentence to make it clear that it is not a statistical analysis (L453-L456).

9. "*L471 ... Cas9 toxicity. Cas12 might be more suitable for some Phytophthoras as demonstrated by Mendoza et al., https://doi.org/10.1094/MPMI-05-23-0072-SC*"
   - This is a very good point, thank you. We have now added an additional two sentences to our Discussion (L507-L510) to add a reference to the Cas9 toxicity in Phytophthoras.

Reviewer #2 Comments:

1. "*A few suggestions and comments are in the file attached.*"
   a. Reviewer #2 suggested that "ambient temperature" be changed to "room temperature" in the methods.
      - We have now changed all instances of "ambient temperature" to "room temperature"; there were eight total changes.

   b. Reviewer #2 found an error in two of our figure captions where the word "from" had been repeated.
      - Thank you for catching this; we have now deleted the extra "from" in the Fig 2 caption (L296). The other double "from" was in S9 Fig, which has now been removed in the revised manuscript.

2. "*Moreover, I think there are too many results in the supplementary materials, so it is better to move to the main text.*"
   - Thank you for this feedback. In response to this, we removed S9 Fig from the supplementary materials and combined the results into Fig 2 of the main manuscript. We also edited Table 3 to include more results from the transformations, and added text to the Results (L377-L380). Finally, to reduce the overall number of supplementary files, we combined S5-S8 Figs into a single revised S5 Fig.

3. "*Check the references and rightly put them as requested by the journal.*"
   - See response to Editor Comment #6 above.

4. "*The English can be improved.*"
   - We feel that the manuscript is written in clear and concise language and cannot see any areas that would require major revisions to improve the English. If the Editor disagrees, we will need more specific instructions as to what can be improved.

Author-initiated revisions:
Minor grammatical, clarification, citation, and reference edits were made to the manuscript to account for revisions made in response to editor and reviewer comments. All areas where changes were made are highlighted with Track Changes or are noted with comments in the revised manuscript. Table numbers were modified to account for the missing Table 2 in the original manuscript. Minor edits were made to S1 Table to account for the changes made to the PCR results in response to Reviewer #1's concerns. Finally, because the gels for Figures 2 and 4 both included a negative water control, and we were re-running the PCR and gel for Figure 2, we also re-ran the gel for Figure 6 to include the negative water control to make the three figures more consistent with each other for the final manuscript.
